# Investigating the causal role of MRE11A p.E506* in breast and ovarian cancer

**DOI:** 10.1038/s41598-021-81106-w

**Published:** 2021-01-28

**Authors:** Islam E. Elkholi, Massimo Di Iorio, Somayyeh Fahiminiya, Suzanna L. Arcand, HyeRim Han, Clara Nogué, Supriya Behl, Nancy Hamel, Sylvie Giroux, Manon de Ladurantaye, Olga Aleynikova, Walter H. Gotlieb, Jean-François Côté, François Rousseau, Patricia N. Tonin, Diane Provencher, Anne-Marie MesMasson, Mohammad R. Akbari, Barbara Rivera, William D. Foulkes

**Affiliations:** 1 Montreal Clinical Research Institute (IRCM), Montreal, QC Canada; 2 Molecular Biology Programs, Université de Montréal, Montreal, QC Canada; 3 Gerald Bronfman Department of Oncology, McGill University, Montreal, QC Canada; 4 Department of Human Genetics, McGill University, Montreal, Canada; 5 Lady Davis Institute, The Jewish General Hospital, Montreal, Canada; 6 Cancer Research Program, The Research Institute of the McGill University Health Centre, Montreal, Canada; 7 Molecular Mechanisms and Experimental Therapy in Oncology Program, Bellvitge Biomedical Research Institute (IDIBELL), L’Hospitalet de Llobregat, 3a planta/Gran Via de l’Hospitalet, 199-203, 08908 Barcelona, Spain; 8 Department of Pediatric and Adolescent Medicine, Children’s Research Center, Mayo Clinic, Rochester, USA; 9 Centre de Recherche du Centre Hospitalier, Universitaire de Québec, Québec City, QC Canada; 10 Centre de Recherche du Centre Hospitalier de L’Université de Montréal and Institut du Cancer de Montréal, Montreal, QC Canada; 11 Department of Pathology, Jewish General Hospital, Montreal, Canada; 12 Division of Gynecologic Oncology, Jewish General Hospital, McGill University, Montreal, QC Canada; 13 Segal Cancer Center, Lady Davis Institute of Medical Research, McGill University, Montreal, QC Canada; 14 Department of Anatomy and Cell Biology, McGill University, Montréal, QC Canada; 15 Department of Medicine, Université de Montréal, Montréal, QC Canada; 16 Department of Medicine, McGill University, Montreal, Canada; 17 Division of Gynecologic Oncology, Université de Montréal, Montreal, Canada; 18 Dalla Lana School of Public Health, University of Toronto, Toronto, Canada; 19 Women’s College Research Institute, Women’s College Hospital, Toronto, Canada

**Keywords:** Breast cancer, Gynaecological cancer, Clinical genetics

## Abstract

The nuclease MRE11A is often included in genetic test panels for hereditary breast and ovarian cancer (HBOC) due to its BRCA1-related molecular function in the DNA repair pathway. However, whether *MRE11A* is a true predisposition gene for HBOC is still questionable. We determined to investigate this notion by dissecting the molecular genetics of the c.1516G > T;p.E506* truncating *MRE11A* variant, that we pinpointed in two unrelated French-Canadian (FC) HBOC patients. We performed a case–control study for the variant in ~ 2500 breast, ovarian, and endometrial cancer patients from the founder FC population of Quebec. Furthermore, we looked for the presence of second somatic alterations in the *MRE11A* gene in the tumors of the carriers. In summary, these investigations suggested that the identified variant is not associated with an increased risk of developing breast or ovarian cancer. We finally performed a systematic review for all the previously reported *MRE11A* variants in breast and ovarian cancer. We found that *MRE11A* germline variants annotated as pathogenic on ClinVar often lacked evidence for such classification, hence misleading the clinical management for affected patients. In summary, our report suggests the lack of clinical utility of *MRE11A* testing in HBOC, at least in the White/Caucasian populations.

## Introduction

Ten to twenty percent of breast and ovarian cancer cases, respectively, are hereditary^1,2^. About 25% of hereditary breast and ovarian cancer (HBOC) cases are attributed to pathogenic variants in the highly penetrant *BRCA1/2* genes^3^. This fueled the hunt for other potential predisposition genes. As *BRCA1/2* and *PALB2*, the most established HBOC predisposing genes, act mainly to orchestrate the dynamics of DNA repair, it has been postulated that genes feeding into the same functional circuit are potential breast and ovarian cancer predisposing candidate genes^4^. One of such proposed candidate genes is *MRE11A.* Biallelic pathogenic *MRE11A* germline variants cause the autosomal recessive ataxia-telangiectasia-like disorder (ATLD; OMIM #604391) which presents with a milder course than ataxia-telangiectasia. Together with NBS1/NBN and RAD50, the nuclease MRE11A forms the MRN complex that binds to BRCA1 upon DNA damage to maintain genomic integrity. This notion strengthened *MRE11A*’s candidacy as an HBOC risk gene, prompting its integration in HBOC screening panels. For instance, Castera et al. screened 708 HBOC patients with an NGS focused panel (27 genes including *MRE11A*) and defined 3 truncating variants in *MRE11A*^5^. In another report, Couch et al. screened around 2000 breast cancer patients for germline variants using a 17-gene sequencing panel and defined two truncating variants in *MRE11A*^6^, as well. However, whether *MRE11A* is a true cancer susceptibility gene remains unclear for two main reasons. First, there is no established cancer risk in the MRE11A-deficient ATLD patients. So far, only two siblings with ATLD were diagnosed with lung adenocarcinoma at the ages of 9 and 15, without a family history of cancer^7^. Second, the lack of comprehensive analysis of individual *MRE11A* variants such as nonsense and rare missense variants through population studies back-to-back with molecular analysis. Indeed, the latter could complement the holistic gene-level analyses, such as the one reported in the recent study by LaDuca et al. who demonstrated the lack of association between the *MRE11A* predicted pathogenic variants and increased risk for different cancer types, including approximately 89,000 breast cancer patients^8^.

In this study, we identified the truncating c.1516G > T;p.E506* *MRE11A* variant (rs587781384) in two unrelated French-Canadian women with breast or ovarian cancer following whole-exome sequencing and a broad clinical HBOC panel, respectively. Since, studying founder populations can help in risk estimation and as we have been studying French Canadians (FCs) for some years^9,10^, we thought we could tackle the questionable *MRE11A* HBOC risk candidacy by investigating the molecular genetics of this single truncating variant, which appears to be overrepresented in the FC population of Quebec compared with other populations studied thus far.

## Results

### Identifying the truncating c.1516G > T;p.E506* MRE11A variant in two unrelated HBOC patients

**Figure 1:**
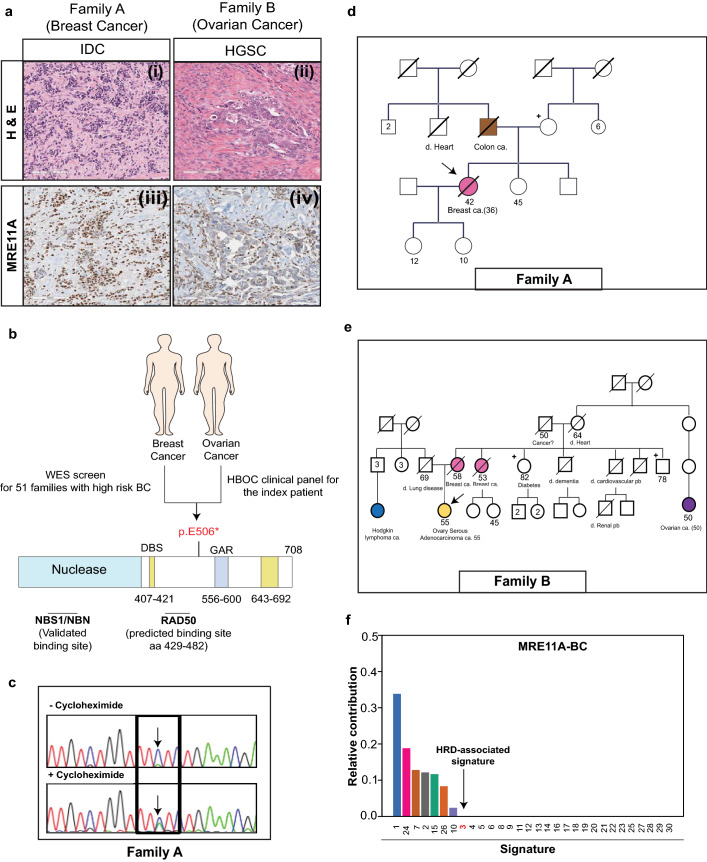
Investigating the c.1516G > T;p.E506* *MRE11A* variant in two unrelated HBOC patients of French-Canadian origins. (**a**) (i) and (ii) H&E staining of the two probands’ breast (invasive ductal carcinoma; IDC) and ovarian tumors (high grade serous carcinoma; HGSC), respectively. (iii) and (iv) Immunohistochemistry staining demonstrating the MRE11A protein expression levels in the two probands’ tumors. (**b**) Schematic representation of the identified variant’s position within the MRE11A protein. (**c**) Cycloheximide chase assay showing that the identified variant undergoes nonsense-mediated mRNA decay, as denoted by the arrow, in the Family A proband-derived lymphoblastoid cell line. (**d**,**e**) Pedigrees of Family A and B, respectively. Carriers of c.1516G > T;p.E506* MRE11A are indicated by + and probands are indicated by arrows. (**f**) Contribution of the different COSMIC single base substitution (SBS) mutational signatures. The SBS3, the Homologous Repair Deficiency (HRD)-associated mutational signature, is indicated by an arrow. (Pedigrees were generated by the *PhenoTips* opensource https://phenotips.com/index.html).

A 36 year-old woman was referred to the cancer genetics clinic with a diagnosis of invasive breast ductal carcinoma. Her clinical test for germline pathogenic mutations in *BRCA1/2* and *PALB2* resulted negative. We then performed whole exome sequencing (WES) in DNA from her peripheral blood mononuclear cells (pbmc) and identified a germline truncating variant c.1516G > T;p.E506* in the *MRE11A* gene (Methodology, and Table S1). A few months later, a 55 years-old woman diagnosed with ovarian high-grade serous carcinoma tested negative for *BRCA1/2* and *PALB2* in a broad HBOC clinical panel (Ref.^11^ for the panel), however the same truncating variant c.1516G > T;p.E506* in *MRE11A* was identified (Fig. 1a,b). Both probands were of a French-Canadian ancestry. The c.1516G > T;p.E506* variant was previously reported in three *BRCA1*/*2* negative breast and/or ovarian cancer patients^6,12,13^ and twice in ATLD cases on ClinVar. The variant also has a gnomAD frequency of 3.19E-05 and 4E–05 in the UK biobank European-ancestry individuals^14^. Given the variant’s low frequency in public databases, we hypothesized that this rare *MRE11A* variant might be a candidate founder pathogenic variant (PV) for HBOC in FCs.

### Further genetic analysis of the c.1516G > T;p.E506* MRE11A variant

During the discovery phase of the project, a segregation analysis was done on the available members of the two families. In family A, the unaffected mother of the proband resulted to be a carrier of the variant. In family B, two healthy relatives of the proband (a maternal aunt and uncle) tested negative (Fig. 1d,e).

To address the possibility of this variant being a founder PV in FCs, we performed a case–control study including 1925 breast, 341 ovarian, and 367 endometrial cancer patients, in addition to 2,287 adult controls and 1932 newborns, all of FC origin (Table 1). The c.1516G > T;p.E506* variant was not observed in any of the cancer patients, but it was found in 4 control subjects (0.002%; 2 males and 2 females). This suggested that c.1516G > T;p.E506*, which is the most reported likely pathogenic variant in *MRE11A* on ClinVar, is overrepresented in the FC population, but is unlikely to be associated with a risk of breast or ovarian cancer.

**Table 1:** Case–control study for the C.1516G > T;p.E506* MRE11A variant in French-Canadians.

		Cases			Controls		
		BC	OC	EC	Adult FC controls Unselected	Adult FC control Selected	FC Newborns
**Number of persons studied**		1925	341	367	396	1891 (50% Females)	1932
		< 70 y/o: 512 Medium Risk: 1201 High Risk: 212					
MRE11A p.E506* carriers	Male	0/5	NA	NA	0	2/946	
	Female	0/1920	0/341	0/341	0	2/945	
	Total (%)	0/1925	0/341	0/341	0/396	4/1891 (0.002%)	1 (0.00052%)

*BC* breast cancer, *OC* ovarian cancer, *EC* endometrial cancer, *MR* moderate risk, *HR* high risk.

Nonsense-mediated mRNA decay (NMD) of alleles harboring a truncating mutation has been postulated as a molecular mechanism by which tumor suppressor genes get inactivated^15,16^. According to the recently-established four rules of NMD, the c.1516G > T;p.E506* variant is predicted to trigger NMD^15,16^. Hence, we carried out a nonsense-mediated mRNA decay (NMD) assay, where a lymphoblastoid cell line derived from the proband of Family A was treated either with cycloheximide to inhibit mRNA degradation or DMSO as a control, as previously described^17^. This assay confirmed that the c.1516G > T;p.E506* variant can indeed trigger NMD as expected (Fig. 1c).

We next investigated the presence of second pathogenic alterations in the tumors of the two probands. No other somatic mutations in *MRE11A* was found by Sanger sequencing and none of the tumors showed any signs of loss-of-heterozygosity, an expected genetic event in the course of developing tumors through inactivating tumor suppressor genes. We then performed WES in the breast tumor and mutational signature analysis^18^ showed that the breast tumor lacked the characteristic Homologous Recombination Repair Deficiency (HRD) mutational signature, MutSign3, arguing against the involvement of c.1516G > T;p.E506* in breast cancer tumorigenesis (Fig. 1f). DNA quality of the ovarian tumor did not meet requirements for WES. At the tumor level, immunohistochemistry staining demonstrated that the breast tumor sample retained MRE11A protein expression (Fig. 1a). In contrast, despite the lack of additional somatic hits in the *MRE11A* gene, the ovarian tumor showed loss of MRE11A protein expression (Fig. 1a). However, the mechanism is uncertain given that the wild-type allele is retained at the DNA level. Altogether, these results indicated that the c.1516G > T;p.E506* variant does not increase the risk for breast and ovarian cancer in FCs.

### Review of the reported MRE11A variants in breast and/or ovarian cancer

**Figure 2:**
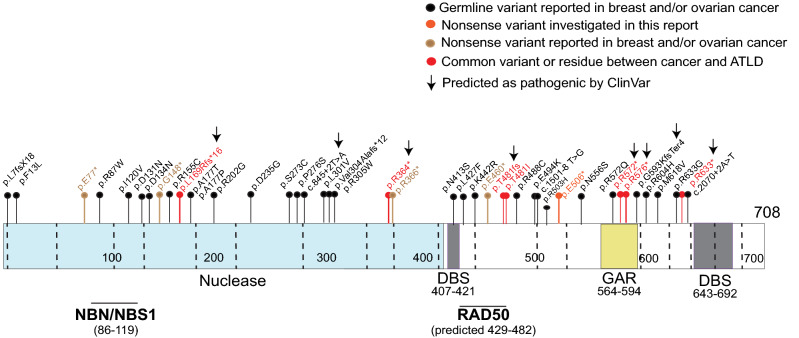
Schematic representation of the previously reported 43 MRE11A germline variants in breast and ovarian cancer. Eight variants are classified as pathogenic on ClinVar (denoted to by arrows). Five variants in addition to a residue were commonly reported between ATLD and cancer patients (red circles/sticks). *GAR* glycine and arginine rich motif, *DBS* DNA-binding site.

To further complement our investigation and to generally address the initial question of the debatable pathogenicity and clinical utility of *MRE11A* testing in HBOC, we performed a systematic review of all previously reported *MRE11A* germline variants in breast and ovarian cancer patients. A total of 43 distinct *MRE11A* germline variants have been reported in such patients over the past 17 years (Table S2). Eight were annotated as pathogenic variants in ClinVar. The main criteria for pathogenic annotation in ClinVar included being a truncating allele, undetectable/absent in control populations, and/or being reported in ATLD or in HBOC patients. Five of those variants had been published or reported in ClinVar as commonly altered among ATLD and cancer cases (Fig. 2 and Table S2).

The c.1516G > T;p.E506* variant was classified as pathogenic in two ATLD cases. However, it had conflicting classifications in hereditary cancer cases. One submission annotated the variant as pathogenic and another as a variant of uncertain significance.

Altogether, these observations demonstrate a clear limitation in the pathogenicity classification of the *MRE11A* germline variants on ClinVar, particularly as applied to HBOC.

## Discussion

Advancement in DNA sequencing technologies has enabled the parallel testing of up to 100 genes within multiple-gene sequencing panels^19^. However, whether all the tested genes are true risk/predisposition genes is questionable. Two main criteria were previously proposed to guide indication for clinical gene screening: clinical validity and utility^20,21^. Both require the candidate to possess genetic variants that are associated with a phenotype (i.e. increased risk of developing cancer) and to predict the clinical outcome and help guide patient management. One of the questionable genes is *MRE11A* that has been included in HBOC gene panels for over a decade mainly for its BRCA1-closely related DNA repair molecular actions.

In this report, using a well-studied founder population, we provide strong support for the notion that protein truncating germline variants in *MRE11A* are not associated with an increased risk of breast, ovarian, or likely, endometrial cancer. This is in accordance with a recently published retrospective study that analyzed the associated risk with predicted pathogenic variants of 32 cancer predisposition genes, including *MRE11A*, in 165,000 persons^8^. Seventy two percent (~ 120,000 persons) had cancer, of which ~ 89,000 were women with breast cancer, ~ 13,000 with ovarian cancer, in addition to ~ 5500 women with endometrial cancer. The team identified 140 predicted pathogenic MRE11A variants in total in different cancers (90 in breast cancer, 11 in ovarian cancer, and 5 in endometrial cancer). The authors did not identify a significant association between the *MRE11A* predicted pathogenic variants and any cancer phenotype, including breast, ovarian, and endometrial cancer^8^, suggesting a lack of *MRE11A* screening utility in HBOC and likely endometrial cancer patients. In another recent study with a similar objective ^22^, Lee et al. investigated the association between the frequently included genes in genetic screening panels, including *MRE11A*, and familial breast and ovarian cancer. The authors used a point-based curation system that takes into account genetic and experimental evidence to score the gene association with the tested cancer type. The categories included Definitive, Strong, Moderate, Limited, Refuted, Disputed, or No Reported Evidence for association. *MRE11A* association with familial breast and/or ovarian cancer was disputed, denoting to the conflicting data and views of its association with the cancer. Our findings, taken together with the recent report of LaDuca et al.^8^ call for re-evaluating the *MRE11A* classification in HBOC. Additional investigations targeting rare missense variants in *MRE11A*, such as those listed in Table S2 and shown in Fig. 2, and their possible association with HBOC are, however, needed to complete the picture for *MRE11A* and HBOC risk. Additionally, limited by the relatively low number of tested endometrial cancer patients in the current report, we cannot completely exclude a role for *MRE11A* truncating variants in the development of endometrial cancer. It is also worth noting in this context that most of the studies focused on Caucasian populations and rarely focused on Black populations. Importantly, Hart et al*.* recently reported threefold and twofold increase in *MRE11A* variants prevalence in ovarian and breast cancer, respectively, in Black population versus Non-Hispanic Whites^23^.

Our systematic review highlights some of the gaps in our current knowledge of the validity and utility of MRE11A testing. Classifying the *MRE11A* ATLD and truncated variants as pathogenic in HBOC should be supported, as previously recommended^24^, by further clinical evidence (e.g., population and/or segregation studies) and/or functional studies to assess the possible associated MRE11A function perturbation. Notably, the truncating c.1516G > T;p.E506* variant, investigated here, was not associated with HRD (Fig. 1f), an expected consequence of a disfunctional MRE11A.

MRE11A variants are, however, associated with a clinically important cancer-related phenotype: clonal hematopoiesis. Two independent studies have found that heterozygous rare variants in MRE11A are subject to copy-neutral loss of heterozygosity, which is strongly associated with clonal hematopoiesis^25,26^. These clones become much more evident with age, such that clonal hematopoiesis affects 40% of Japanese persons over age 90 years. Therefore, MRE11A variants deserve further attention as they are associated with adverse health outcomes.

In conclusion, we report the detailed investigation of a truncating variant in the candidate breast and ovarian cancer susceptibility gene, *MRE11A*. Our findings, taken together with other recently published studies, show that truncating variants in *MRE11A* are not a cause of breast or ovarian cancer. Based on the current evidence, testing for *MRE11A* variants should not be offered to women with, or at risk for, breast or ovarian cancer.

## Patients and methods

### Patients and samples

In total, 2633 cancer patients and a control group of 2287 adults and 1932 newborns of French-Canadian ancestry were included in this study, as explained in detail in Table 1 and the study population section.

### Ethics statement

The initial two families and control subjects were recruited following approval of the Institutional Review Board of the Faculty of Medicine of McGill University (IRB 2019-5465) and the Jewish General Hospital (2016-397), respectively and written informed consent was obtained.

Newborn blood left-over after delivery was collected at the Hôpital Saint-François d’Assise maternity unit between 1996 and 2003. DNA was extracted and a biobank with over 7000 samples was constituted. These samples were made anonymous and unlinked. About 15% of the samples were randomly excluded from the collection in order to preserve even further the anonymity of the samples because any woman having given birth in this hospital during that period has a 15% chance of not being part of the studied samples. The Hôpital Saint-François d’Assise Clinical Research Ethics Committee approved the research project. For the present study a subset of n = 1932 samples from the collection was randomly selected based uniquely on amount of DNA available as it is not possible to identify the subset of samples included in the study, sex information is not available.

Samples from healthy controls (1891 individuals) were obtained through CARTaGENE biobank^27^ (https://www.cartagene.qc.ca/en/home). Ovarian and endometrial cancer patients in addition to the under 70 years old breast cancer patients’ group were obtained through the Banque de tissus et de données of the Réseau de recherche sur le cancer of the FRQS. All participants were recruited in compliance with the second edition of the Canadian Tri-Council Policy Statement of Ethical Conduct for Research Involving Humans and Eligible Persons or Designates and signed a consent form in accordance with the Institutional Review Board approvals. All methods were performed in accordance with the relevant guidelines and regulations.

### Index patients and their families

The proband of family A was diagnosed at the age of 36 with invasive ductal carcinoma. This patient is part of a 51-high risk BRCA1/2 negative BC patients study previously carried out by our team^28^. The patient had been screened for germline variants by whole exome sequencing (WES) revealing the presence of a likely pathogenic variant in MRE11A (c.1516G > T;p.E506*) (Fig. 1a,b). Proband of Family B was a 55-year old woman diagnosed with a high-grade serous carcinoma (HGSC) that was referred to the cancer genetics clinic from the gynecology clinic at the Jewish General Hospital (Montreal, Canada) for genetic testing (Fig. 1a,b). The patient had a family history strongly suggestive of hereditary breast and ovarian cancer (HBOC) (mother and aunt diagnosed with breast cancer). A clinical HBOC screening panel identified the same nonsense MRE11A c.1516G > T;p.E506*variant independently found in the proband of family A. Both women were of French-Canadian ancestry.

### Study population (case–control study)

During the validation phase for this study, 2633 cancer patients and a control group of 2287 adults and 1932 newborns of French-Canadian ancestry were studied (Table 1). In detail, 1925 BC including 212 (207 women, 5 men) high-risk cases (age, 19–73; average, 45) in addition to 1201 (all women) moderate risk BC cases (age, 25–68; average, 48) that we previously described in detail^9^ and a group of 512 BC diagnosed under 70 years of age^29,30^. Briefly, the risk classification of the BC patients depended mainly on the age of diagnosis and presence of family history (high risk = early onset and/or strong family history; moderate risk is defined as age at diagnosis < 50 years or ≥ 50 years but with affected first- or second-degree relatives). The study included 341 HGSC^31^ (age, 36–87; median age 61 years) and 367 endometrial cancer patients (age, 21–92; average 62) of French-Canadian origins^9^. In parallel, 1891 healthy adults with no personal history of cancer or diagnosed cancer in 1st degree relative were recruited as controls. Those controls were aged 40 to 70 that were ascertained between 2009 and 2015 through the CARTaGENE biobank^27^ (https://www.cartagene.qc.ca/en/home) living in the Greater Montreal area and their FC ancestry was defined as French as their first language with their four grandparents being of Canadian origin^30^. Additionally, 396 control subjects were recruited as unselected group^9^. Finally, to assess the prevalence of the identified variant in FCs, we screened 1932 newborns of French-Canadian ancestry^28^.

### Whole exome sequencing (WES)

The proband A was among a 51-high risk BC patient series we previously screened for germline variants in genomic DNA extracted from peripheral blood leukocytes as previously outlined in detail^28^. Breast cancer from the proband studied here was subjected to WES that was performed at the McGill University and Genome Quebec Innovation Centre (MUGQIC). Breast cancer FFPE-derived DNA sample underwent exome capture (Nextera Rapid Capture Exome Kit), followed by 100 bp paired-end sequencing on Illumina HiSeq 2500 (details in Ref^26^). Bioinformatics analysis of exome sequencing data was performed using our WES pipeline as previously described^9,32–34^. In brief, alignment of sequenced reads to the reference genome (hg19) was performed using BWA^35^ (v. 0.5.9). Subsequently, the Genome Analysis Toolkit (GATK) was used to perform local realignment of reads around small insertions and deletions (indels) and to assess capture efficiency and coverage for all samples. The coverage statistics are listed in Table 2. Likely pathogenic variants in 152 known cancer susceptibility genes from Huang et al.^36^ and in 468 cancer panel gene list elaborated by the MSK-IMPACT were selected. Candidate somatic mutations were subjected to several filtering steps and eliminated if they fulfilled any one of the following criteria: (1) genomic position of variant covered by < 5-reads, (2) < 5 reads support the alternative variant, (3) variant has allelic ratio < 10% for SNVs or < 15% for indels, (4) variant has allele frequency > 0.001 in TCGA-ExAC databases (release 0.3 2016-01-13) or seen as homozygote in ExAC database (build). (5) missense variants that were not predicted to be disease causing by 3 out of 6 bioinformatic algorithms (SIFT, PolyPhen, MutationTaster, Revel, MCAP and CADD)^37–42^.

**Table 2:** Exome sequencing coverage summary*.*

Sample	Mean coverage	%CCDS bases ≥ 5× coverage	%CCDS bases ≥ 10× coverage	%CCDS bases ≥ 20× coverage	%CCDS bases ≥ 50× coverage
Blood-family A	122	96	95	91	75
Breast cancer-family A	67	97	93	84	54

*CCDS* coverage of consensus coding sequence.

### Mutational signature

SomaticSignatures, a Bioconductor package, was used to analyze all the filtered synonymous and nonsynonymous somatic mutations. Briefly, 96 trinucleotides mutational context [A|C|G|T] [C > A|C > G|C > T|T > A|T > C|T > G] [A|C|G|T] were extracted and compared with the current known set of COSMIC single base substitution signatures (SBS) listed by the Sanger Institute^18^ https://cancer.sanger.ac.uk/cosmic/signatures/SBS/index.tt. The Homologous Repair Deficiency (HRD) mutational signature corresponds to SBS3 as denoted in the text and Fig. 1f.

### MRE11A c.1516G > T genotyping

Moderate-risk breast cancer samples were genotyped using a TaqMan SNP Genotyping assay. For the HGSC samples, DNA from peripheral blood lymphocytes (n = 305), ovarian tumours (n = 34) or ascites (n = 2) were genotyped using The Custom TaqMan SNP Genotyping Assay protocol performed as previously described^43^. Using a similar pipeline to our previous work^28^, we genotyped samples from the below 70 years old breast cancer group (n = 512), newborns of French Canadian ancestry (n = 1932) in addition to the healthy control adults (CARTaGENE biobank; n = 1891) for MRE11A c.1516G > T; p.E506* allele using the iPLEX MassARRAY platform (Sequenom) at the McGill University and Genome Quebec Innovation Centre (MUGQIC). A high-resolution melting (HRM) assay was used to test all the remaining patient and control samples included in this study as previously described^9^. For validations, Sanger sequencing was used, and chromatograms were aligned with the NM_005591.4 RefSeq transcript. Probes and primers sequences are available upon request.

### Databases and software used in this report

*gnomAD*. We interrogated the gnomAD database^44^ to assess the frequency of the p.E506* MRE11A variant and its prevalence in the reported ethnicities. gnomAD v2.1.1 was last accessed on 30 April 2020 and the MRE11 p.E506* variant (variant ID:11-94189489-C-A; GRCh37) prevalence was detected as detailed in the text.

The *PhenoTips* software was used to generate pedigrees (https://phenotips.com/index.html). The *ProteinPaint tool*^45^* from* https://proteinpaint.stjude.org/ was used for elaboration of Fig. 2.

### Immunohistochemistry

Formalin-fixed, paraffin-embedded (FFPE) samples from the breast and ovarian tumors were studied by IHC. MRE11A (Abcam #ab214, 1:1500) was optimized in a Ventana machine following recommended protocols. MRE11A expression and localization was analyzed by a pathologist (OA) independently for diagnosis and mutational analysis. Aperio ImageScope software was used to obtain the images.

### NMD assay

A lymphoblastoid cell line was established from a blood sample from the proband of Family A. The cells were cultured using RPMI medium. For assessing whether the mutant allele undergoes NMD, cells were plated in 6 well plates, and either treated with cycloheximide (28 ug/ml)^17^ or (DMSO) for 3 h. At the experimental endpoint, RNA was extracted from both the treated or untreated cells using Trizol and RT-PCR was performed to obtain cDNA. cDNA was then subjected to sanger sequencing.

### MRE11A germline variants literature review

We did a literature review for the reported MRE11A germline variants as of May 13 2020. We found 21 studies reporting different variants of MRE11A in mainly breast and ovarian cancers over the past 17 years (from 2003 to 2020). One study was excluded as it did not focus on HBOC. All the related info and references are included in Table S2.

## Supplementary Information

Supplementary information.
